# Use of In-Clinic Diagnostic Kits for the Detection of Seropositivity to *Leishmania infantum* and Other Major Vector-Borne Pathogens in Healthy Dogs

**DOI:** 10.3390/pathogens12050696

**Published:** 2023-05-11

**Authors:** Simone Morelli, Anastasia Diakou, Antonio Frangipane di Regalbono, Mariasole Colombo, Giulia Simonato, Angela Di Cesare, Alessandra Passarelli, Carlo Pezzuto, Zoe Tzitzoudi, Alessandra Barlaam, Melissa Beall, Ramaswamy Chandrashekar, Nikola Pantchev, Donato Traversa

**Affiliations:** 1 Department of Veterinary Medicine, University of Teramo, 64100 Teramo, Italy; mcolombo@unite.it (M.C.); adicesare@unite.it (A.D.C.); dtraversa@unite.it (D.T.); 2 School of Veterinary Medicine, Faculty of Health Sciences, Aristotle University of Thessaloniki, 54124 Thessaloniki, Greece; diakou@vet.auth.gr (A.D.); zoimaria.vet@gmail.com (Z.T.); 3 Department of Animal Medicine, Production and Health, University of Padua, 35020 Legnaro, Italy; antonio.frangipane@unipd.it (A.F.d.R.); giulia.simonato@unipd.it (G.S.); 4 Clinica Veterinaria Città di Bari, 70125 Bari, Italy; alessandra_pax@hotmail.com; 5 Ambulatorio Veterinario Pezzuto Carlo/Piano Noemi, 86010 Campobasso, Italy; pezzutoepiano@gmail.com; 6 Department of Agriculture, Food, Natural Resources and Engineering, University of Foggia, Via Napoli 25, 71121 Foggia, Italy; alessandra.barlaam@unifg.it; 7 IDEXX Laboratories Inc., Westbrook, ME 04092, USA; Melissa-Beall@idexx.com (M.B.); Chandra-Chandrashekar@idexx.com (R.C.); 8 IDEXX Laboratories, 70806 Kornwestheim, Germany; nikola-pantchev@idexx.com

**Keywords:** vectors, dogs, *Leishmania*, diagnosis, diseases

## Abstract

Canine Vector-Borne Diseases (CVBDs) are widespread in Europe and enzootic in many other countries. Though severe illnesses may occur, dogs living in enzootic areas often show vague or no clinical signs of CVBDs. Undiagnosed infections/co-infections in subclinically infected animals favor the spread of CVBDs and increase the risk of transmission to other animals and, in some cases, humans. This study has evaluated the exposure of dogs living in key enzootic countries, i.e., Italy and Greece, to major CVBDs via the use of in-clinic diagnostic kits. Overall, 300 privately owned dogs without/with single mild clinical signs living in different regions of Italy (n. 150) and Greece (n. 150) were included in the study. As part of a clinical examination, a blood sample was collected from each dog and subjected to two serological rapid tests, i.e., the SNAP^®^ 4Dx^®^Plus (IDEXX Laboratories Inc.) for the detection of antibodies against *Ehrlichia* spp., *Anaplasma* spp., *Borrelia burgdorferi* s.l. and *Dirofilaria immitis* antigen and the SNAP^®^ *Leishmania* (IDEXX Laboratories Inc.) for the detection of antibodies against *Leishmania infantum*. In all, 51 dogs (17%; 95% CI 12.9–21.7) were seropositive to at least 1 pathogen, i.e., 4 in Italy (2.7%; 95% CI 1.4–13.1) and 47 in Greece (31.3%; 95% CI 24–39.4). *Dirofilaria immitis* antigens were found in 39 dogs (13%; 95% CI 9.4–17.3), while antibodies against *Ehrlichia*, *Anaplasma* and *Leishmania* were detected in 25 (8.3%; 95% CI 5.5–12.1), 8 (2.7%; 95% CI 1.2–5.2) and 5 (1.7%; 95% CI 0.5–3.8) dogs, respectively. None of the dogs tested seropositive for *B. burgdorferi* s.l. Statistical analyses were performed to evaluate associations between exposure to CVBDs and possible risk factors. The present results indicate that dogs living in enzootic areas may be seropositive for one or more CVBDs in absence of clinical signs. Rapid kits are among first line tools for the detection of CVBDs in clinical settings, as they are cost-effective, straightforward and quick to use. Also, in-clinic tests used herein allowed detection of co-exposure to CVBDs investigated.

## 1. Introduction

In enzootic areas, dogs are exposed to the bite of arthropods able to transmit several pathogens, representing a primary threat to animal and human health [1,2,3,4,5,6]. In Europe, the most important agents of Canine Vector-Borne Diseases (CVBDs) include the tick-borne bacteria *Ehrlichia canis*, *Anaplasma platys*, *Anaplasma phagocytophilum*, *Borrelia* spp. and *Rickettsia* spp., the nematode *Dirofilaria immitis* and the protozoans *Leishmania infantum* and *Babesia* spp., transmitted by mosquitoes, sandflies and ticks, respectively [7,8,9,10,11,12,13]. European regions have suitable environments for the occurrence of diseases transmitted by invertebrates and, among countries, Italy and Greece are epizootiologically significant areas for many CVBDs [11,14,15,16,17,18,19,20,21].

Infected dogs may display signs of varying severity, from none or mild to severe and life-threatening clinical manifestations and abnormalities [9,11,12]. However, especially in enzootic areas, infected dogs do not often show significant clinical signs and may still act as a source of infection for the vectors. Though the role of subclinically infected dogs as carriers of certain CVBPs should be further investigated, undetected infections may lead to (i) underdiagnosis, which could prevent the detection of subclinical laboratory alterations, e.g., early kidney disease, anemia, thrombocytopenia, increased CRP (especially in co-infections), (ii) underestimation of disease prevalence and (iii) spread of CVBDs in both enzootic and free regions. On the other hand, dogs are epidemiological sentinels in enzootic areas and continuous serological monitoring is useful to assess their exposure to major CVBDs [4,11,22]. In fact, the detection of seropositive dogs may assist in investigating their potential role as sources of infection for vectors (seropositive dogs are not a certain source of infection, especially for tick-borne bacteria, as seropositivity may indicate a past exposure rather than a current infection), provide information on their clinical assessment and indicate the risk for animals and humans. The epidemiological history and the environment where dogs live should be taken into account and the persistence and the ability to cause subclinical infections are variable among pathogens.

Various diagnostic techniques are used to assess the serological status of canine populations living in regions enzootic for CVBDs. While assays that quantitatively evaluate the seropositivity and the antibody titers in exposed/infected animals (e.g., IFAT, ELISA) are typically applied on large-scale surveys, individual diagnosis in the clinic often requires the use of rapid, cost-effective and straightforward qualitative or semi-quantitative tests. Also, some specific-peptides-based rapid kits allow the detection of co-exposure to different pathogens, compared to other serological methods that in many cases are based on the use of whole cells. In addition to the usefulness for preliminary screening and the aid in diagnosis for individual animals, the same rapid tests may be used also in epizootiological studies.

In this survey, two in-clinic rapid kits were used to evaluate apparently healthy dogs living in enzootic regions of major CVBDs, to provide further surveillance on the exposure to CVBDs in key enzootic countries and data for continued refinement of control programs, aiming to protect animal and human health.

## 2. Materials and Methods

### 2.1. Study Design and Sampling

**Figure 1:**
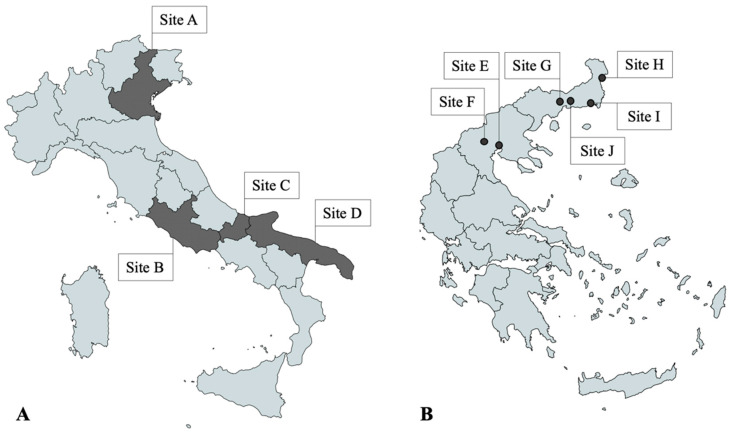
(**A**) Study sites in Italy: regions of Veneto (Site A), Lazio (Site B), Molise (Site C), Puglia (Site D); (**B**) Study sites in Greece: cities of Thessaloniki (Site E), Pella (Site F), Xanthi (Site G), Didimoticho (Site H), Alexandroupoli (Site I), Komotini (Site J). Dogs in Site C were included only during timeframe 1, while dogs from D were included during timeframe 2.

A total of 300 privately owned dogs living in different regions of Italy and Greece were included in the study. In Italy, 50 dogs were selected in Northern (Site A), Central (Site B) and Southern regions (Sites C and D) each. In Northeastern Greece, 25 dogs from each of the six selected sites (Sites E–J) were included in the study (Figure 1). The sampling was divided into two windows within 2022, with half of the dogs included between February and April (timeframe 1—TF1) and the other half between May and September (timeframe 2—TF2).

The survey was conducted in the framework of routine medical checks coordinated by local veterinarians and dogs were selected based on the following criteria: (i) willingness of the owners to monitor the health status of their dogs; (ii) dogs living in areas enzootic for CVBDs; (iii) dog age that permitted at least one vector season experienced; (iv) absence of clinical diseases compatible with any of the major CVBDs under study.

Prior to enrollment, each dog was subjected to a physical examination to evaluate the presence of clinical signs possibly related to CVBDs. For every single clinical sign, a score ranging from 0 to 3 was assigned, as follows: 0 (normal status or absent), 1 (mild), 2 (moderate) and 3 (severe). A clinical score was calculated for each dog based on the sum of the scores of each clinical sign and each dog was categorized as follows: clinically healthy dog (score 0–5), mild disease (score 5–30), moderate disease (score 31–55), severe disease (score 55–75). Only clinically healthy dogs were included in the study.

Signalment and anamnesis, including data on age, sex and breed, were recorded for each dog. A consent form was signed by the owner before blood collection, which was performed via venipuncture of jugular, cephalic or saphenous veins. The blood was transferred into a tube without anticoagulant and centrifuged after clot formation to separate serum and then tested immediately.

### 2.2. Study Animals

Overall, 124 (41.3%) dogs included were male and 176 (58.7%) were female. A total of 36 dogs (24%) enrolled in Italy were ≤2 years old and 114 (76%) were >2 years old. In Greece, 42 (28%) and 108 (72%) were ≤2 years old and > 2 years old, respectively. Among the enrolled dogs, 174 (58%) were crossbred and 126 (42%) were purebred dogs.

### 2.3. Serological Tests and Statistical Analysis

Each serum sample was subjected to two different serological examinations performed by the veterinarians according to the manufacturer’s instructions:

- SNAP^®^ 4Dx^®^ Plus Test (IDEXX Laboratories, Inc., Westbrook, ME, USA) for the detection of *Dirofilaria immitis* circulating antigen (Sensitivity 98.9%, Specificity 99.3%) and of antibodies against specific peptides of *Anaplasma phagocytophilum* (Sensitivity 93.2%, Specificity 99.2%), *Anaplasma platys* (Sensitivity 89.2%, Specificity 99.2%), *Ehrlichia canis* (Sensitivity 97.8%, Specificity 92.3%), *Ehrlichia ewingii* (Sensitivity 96.5%, Specificity 93.9%) and the C6-peptide of *Borrelia burgdorferi* s.l. (Sensitivity 96.7%, Specificity 98.8%) [23];
- SNAP^®^ *Leishmania* Test (IDEXX Laboratories, Inc., USA) (Sensitivity 91.1–93.4%, Specificity 99.2–100%) for the detection of antibodies against *L. infantum* [24].

The GraphPad Prism 9 Software was used for the statistical analysis, based on Fisher’s exact test to evaluate the presence of significant associations (*p* < 0.05) between possible risk factors and exposure to CVBDs. Possible risk factors (i.e., presence of clinical signs, age, sex, breed, timeframe) were evaluated with a binomial logistic regression with a strength measured using the Odds Ratio (OR) and a 95% Confidence Interval.

## 3. Results

Fifty-one out of three hundred (17%; 95% CI 12.9–21.7) dogs included in the study were seropositive to at least one CVBD pathogen (Table 1).

Three dogs (2%; 95% CI 0.4–5.7) sampled in Italy—one and two from central and Southern Italy (Sites B and D), respectively—in TF2 were seropositive for *L. infantum*. Also, one dog sampled in Southern Italy in TF2 was seropositive for the *D. immitis* antigen (Table 2). Overall, 47 dogs (31.3%; 95% CI 31.3; 24–39.4) sampled in Greece were seropositive for at least one pathogen, i.e., 4, 7, 5, 17, 6 and 8 in Sites E to J, respectively (Table 3). Of these dogs, 27 (18%; 95% CI 12.2–25.1) were seropositive to only one pathogen, whilst 20 (13.3%; 95% CI 8.3–19.8) were seropositive for more than one pathogen, i.e., 15, 4 and 1 dogs were seropositive for 2, 3 or 4 pathogens, respectively. The highest seroprevalence was found for *D. immitis* (38 dogs, 25.3%; 95% CI 18.6–33.1), followed by *Ehrlichia* spp. (25 dogs, 16.7%; 95% CI; 11.1–23.6). Accordingly, *D. immitis* and *Ehrlichia* spp. were the most-recorded pathogens in dogs with a single seropositive result (19 and 7 dogs, 12.7%; 95% CI 7.8–19.1 and 4.7%; 95% CI 1.9–9.4).

The most frequent combinations in dogs seropositive for 2 or more pathogens were *Ehrlichia* spp. + *D. immitis* (12 dogs, 4%) and *Ehrlichia* spp. + *A. platys/phagocytophilum* + *D. immitis* (4 dogs, 1.3%) (Table 4).

Among the dogs included in the study, 45 (15%; 95% CI 11.2–19.5) displayed at least one clinical sign (though with a total clinical score between 0 and 5) and 5 (11.1%; 95% CI 3.7–24) of them were seropositive for at least one CVBD.

### Statistical Analysis

The Fisher’s exact test revealed three statistically significant factors associated with the seropositivity to at least one CVBD pathogen: age more than 2 years (*p* = 0.0062; OR = 0.3265), crossbred dogs (*p* < 0.0001; OR = 6.769; 95% CI = 2.84–15.36) and sampling during timeframe 2 (*p* = 0.005; OR = 0.3923; 95% CI = 0.2065–0.7454). Being a crossbred dog was a risk factor for seropositivity to CVBDs from the results of the binomial logistic regression (*p* < 0.0001; OR = 5.823; 95% CI = 2.39–14.19). No other statistically significant associations were detected.

## 4. Discussion

This study demonstrates that dogs without or with single, mild clinical signs living in enzootic areas may be seropositive for one or more major CVBDs. All study animals were residing in Mediterranean territories where several epizootiological, biological and ecological drivers may foster their exposure to bites of infected arthropods [11,25,26,27,28,29]. Relevant and interesting differences have been found among Italy and Greece and among sites of these countries in terms of seropositivity to CVBDs in animals with no evidence of clinical signs.

The low seropositivity rates for *L. infantum* and other CVBDs herein detected in Italy has been most likely influenced by the inclusion of urban dogs only, i.e., at lower risk of CVBD exposure, instead of a randomly selected population, as has been described in classical epizootiological or previous studies [18,19,27]. The same accounts for the seropositivity for *Ehrlichia* spp., *A. platys*/*A. phagocytophilum* and *B. burgdorferi* s.l. [19,30,31,32,33]. It should be considered that the seropositivity rates of CVBDs could vary depending on the diagnostic test used [9,34] and overestimation could occur when low specificity, whole-cell or crude antigen-based tests are used [35].

*Dirofilaria immitis* is traditionally considered enzootic in Northern Italy and has recently been spreading southward [29,36,37]. However, the present results support the decrease in the infection rates detected in other studies, which could be attributed to intensive prophylactic measures applied throughout Italy to privately owned dogs [19,36,37,38]. Nevertheless, to the authors’ best knowledge, chemoprevention against *D. immitis* is not yet routinely performed in Southern and Central Italy, where a further increase in seroprevalence could occur in the near future.

Regarding Greece, the results confirm that the studied CVBDs are enzootic in this country and may occur at a relatively high seroprevalence in apparently healthy dogs, particularly since these pathogens can produce subclinical infections that could be associated with diseases detectable only with laboratory analyses, e.g., early kidney disease [39]. The higher seroprevalence in this group was not surprising, as many of the dogs examined in Greece were previously strays, having little veterinary or preventive care prior to the time of the study.

*Leishmania infantum* is enzootic and widespread in all areas of Greece. The present results suggest that the occurrence of infection in owned dogs seems to be decreasing, probably due to the development and application of effective preventive measures (vaccination, insect repellents) [40,41]. With all likelihood, the seroreactivity to *Ehrlichia* spp. herein detected represented exposure to *E. canis*, as this is the only species occurring in dogs in Europe, and due to the vast distribution of its main vector, *Rhipicephalus sanguineus*. Northern Greece historically displays the highest seroprevalence of *D. immitis* compared to the rest of the country [42]. Despite preventive treatments being applied in most owned dogs in Northern Greece [43], a combination of factors, e.g., wetlands, mosquito populations, stray dogs and wildlife abundance (e.g., foxes, jackals, wolves), result in this epizootiological scenario [42,44].

Such a marked difference in the seropositivity rate of CVBD pathogens in dogs from Italy and Greece was not detected in recent studies; by contrast, a similar occurrence of CVBDs was reported [11,30]. The discrepancy is likely due to the geographic area herein studied, i.e., Northern and Northeastern Greece, where CVBDs are significantly more prevalent than in other areas of the country, probably due to environmental drivers [15,40,42], while the previous study was conducted in Aegean islands, where no differences with the seroprevalence rates in Italy were detected [11]. Furthermore, in the present study, in Italy, almost only urban dogs were enrolled, while most of the dogs enrolled in Greece were from towns located in rural areas that lived outdoors, i.e., gardens and yards, and in environments where the presence of vectors and the infection pressure by CVBD pathogens is particularly high. Dogs living in less urbanized areas may be subjected to higher parasitological pressure, due to the higher density of wild reservoir hosts of both arthropod vectors and transmitted pathogens [45].

It should be kept in mind that dogs that tested seropositive for vector-borne pathogens (e.g., *L. infantum*, *E. canis*) and/or that were repeatedly exposed to one or more CVBDs may be clinically healthy for a certain period of time and then develop overt disease, e.g., kidney disease, only in a later stage of the infection [46,47,48]. Clinically healthy dogs have an important role in the maintenance of the circulation of *L. infantum* within canine and human populations, as they are more rarely examined and screened compared to dogs displaying clinical signs compatible with leishmaniosis [49]. This category of dogs plays an important epizootiological/epidemiological role, as they favor the spread of the infection, especially in cases where routine prophylaxis for leishmaniosis is not performed. As an example, unnoticed infections in non-endemic areas may facilitate the circulation of *L. infantum* among vectors and animal hosts.

The results of the statistical analyses should be interpreted in consideration of some features of the study dogs. The correlation between dogs over 2 years of age and the seropositivity to at least one CVBD is likely due to more chances of contact with the vectors than younger dogs. The apparent higher risk of infection in crossbred dogs has been likely influenced by the inclusion of a high number of crossbreeds (n. 120 dogs) in sites of Greece, especially during TF2.

## 5. Conclusions

In conclusion, rapid kits are among the first-line tools for the diagnosis of CVBDs in clinical settings, due to their limited cost and speed of execution and due to the high specificity ensured by the specific peptide targets for antibody detection. In-clinic kits are useful for the diagnosis of *D. immitis* infection (in combination with appropriate microfilariae-based test), for starting an in-depth diagnostic approach in the case of *L. infantum* infection or for steering the diagnosis towards tick-borne diseases in presence of compatible clinical signs. Testing for CVBDs should become routine in clinical settings, as this would allow the detection of subclinical infections that may subsequently exacerbate (especially co-infections), and when dogs are adopted or do not receive regular veterinary preventive care. While rapid kits are useful and easy to use routinely in veterinary practices, other laboratory serological tests (IFAT, ELISA) are often necessary for the confirmation of infection and/or the quantitative assessment of seropositivity. It is important to avoid unnecessary treatments and to set up a proper diagnostic and therapeutic strategy after a careful clinical evaluation by the veterinarian and on a case-by-case basis. Although clinically healthy dogs generally do not need therapy (unless laboratory alterations are present and/or in presence of high antibody titer), knowledge of their parasitological/serological status is necessary to set a proper follow-up and to apply prophylactic measures to limit the spread of the pathogen.

No rapid kit is currently available for the serodiagnosis of other important CVBDs (e.g., *Rickettsia* spp., *Hepatozoon* spp.) and the presence of the latter pathogens can be investigated only using laboratory serological tests (IFAT, ELISA) and/or molecular analyses (PCR). New rapid diagnostic tools would be highly appreciated by veterinarians in clinical settings, as a laboratory diagnosis using IFAT/ELISA or PCR is usually more expensive, possibly reducing the compliance of the owners to perform the investigation, and is more prone to cross-reactions in some cases [50]. Therefore, future studies aimed at (i) comparing the diagnostic performance of rapid kits vs. IFAT/ELISA and (ii) the development of new sensitive and specific rapid tools for the in-clinic diagnosis of CVBDs are advocated [51].

## Figures and Tables

**Table 1:** Overall number and percentage of dogs seropositive for different vector-borne pathogens in the present study. A total of 300 dogs were examined, i.e., 150 dogs for each timeframe (TF): 75 in sites of Italy and 75 in sites of Greece.

*D. immitis* n (%; 95% CI)	*Anaplasma* n (%; 95% CI)	*Ehrlichia* n (%; 95% CI)	*B. burgdorferi* s.l. n (%; 95% CI)	*L. infantum* n (%; 95% CI)	At Least One Pathogen n (%; 95% CI)
**Italy/Greece TF 1**					
11 (7.3; 3.7–12.7)	3 (2; 0.4–5.7)	6 (4; 1.5–8.5)	0	0	16 (10.7; 6.2–16.7)
**Italy/Greece TF 2**					
25 (16.7; 11.1–23.6)	5 (3.3; 1.1–7.6)	19 (12.7; 7.8–19.1)	0	5 (3.3; 1.1–7.6)	35 (23.3; 16.8–30.9)
**Italy/Greece TF 1 + TF 2**					
39 (13; 9.4–17.3)	8 (2.7; 1.2–5.2)	25 (8.3; 5.5–12.1)	0	5 (1.7; 0.5–3.8)	51 (17; 12.9–21.7)

n = number of seropositive dogs; TF = timeframe.

**Table 2:** Number and percentage of dogs seropositive for different vector-borne pathogens enrolled in Italy. In each study site, 25 dogs have been examined, for a total of 75 dogs for each timeframe (TF). Study sites = regions of Veneto (Site A), Lazio (Site B), Molise (Site C), Puglia (Site D).

Site	*D. immitis* n (%; 95% CI)	*Anaplasma* n (%; 95% CI)	*Ehrlichia* n (%; 95% CI)	*B. burgdorferi* s.l. n (%; 95% CI)	*L. infantum* n (%; 95% CI)	At Least One Pathogen n (%; 95% CI)
**TF 1**						
**A**	0	0	0	0	0	0
**B**	0	0	0	0	0	0
**C**	0	0	0	0	0	0
**Total**	0	0	0	0	0	0
**TF 2**						
**A**	0	0	0	0	0	0
**B**	0	0	0	0	1 (4; 0.1–20.3)	1 (4; 0.1–20.3)
**D**	1 (4; 0.1–20.3)	0	0	0	2 (8; 0.9–26.03)	3 (12; 2.5–31.2)
**Total**	1 (4; 0.03–7.2)	0	0	0	3 (4; 0.8–11.2)	4 (5.3; 1.4–13.1)
**TF 1 + TF 2**						
**Total**	1 (0.7; 0.02–3.66)	0	0	0	3 (2; 0.4–5.7)	4 (2.7; 0.7–6.7)

n = number of seropositive dogs; TF = timeframe.

**Table 3:** Number and percentage of dogs seropositive for different vector-borne pathogens enrolled in Greece. In each study site, 25 dogs have been examined, for a total of 75 dogs for each timeframe (TF). Study sites = cities of Thessaloniki (Site E), Pella (Site F), Xanthi (Site G), Didimoticho (Site H), Alexandruopoli (Site I), Komotini (Site J).

Site	*D. immitis * n (%; 95% CI)	*Anaplasma * n (%; 95% CI)	*Ehrlichia * n (%; 95% CI)	*B. burgdorferi* s.l. n (%; 95% CI)	*L. infantum * n (%; 95% CI)	At Least One Pathogen n (%; 95% CI)
**TF 1**						
**E**	4 (16; 4.5–36.08)	1 (4; 0.1–20.3)	1 (4; 0.1–20.3)	0	0	4 (16; 4.5–36.08)
**F**	7 (28; 12.07–49.4)	1 (4; 0.1–20.3)	1 (4; 0.1–20.3)	0	0	7 (28; 12.07–49.4)
**G**	2 (8; 0.9–26.03)	1 (4; 0.1–20.3)	4 (16; 4.5–36.08)	0	0	5 (20; 6.8–40.7)
**Total**	11 (14.7; 7.6–24.7)	3 (4; 0.8–11.2)	6 (8; 3–16.6)	0	0	16 (21.3; 12.7–32.3)
**TF 2**						
**H**	17 (68; 46.5–85.05)	5 (20; 6.8–40.7)	13 (52; 31.3–72.2)	0	1 (4; 0.1–20.3)	17 (68; 46.5–85.05)
**I**	5 (20; 6.8–40.7)	0	2 (8; 0.9–26.03)	0	0	6 (24; 9.3–45.1)
**J**	3 (12; 2.5–31.2)	0	4 (16; 4.5–36.08)	0	1 (4; 0.1–20.3)	8 (32; 14.9–53.5)
**Total**	24 (32; 21.7–43.8)	5 (6.7; 2.2–14.9)	19 (25.3; 16–36.7)	0	2 (2.7; 0.3–9.3)	31 (41.3; 30.1–53.3)
**TF 1 + TF 2**						
**Total**	38 (25.3; 18.6–33.1)	8 (5.3; 2.3–10.2)	25 (16.7; 11.1–23.6)	0	2 (1.3; 0.2–4.7)	47 (31.3; 24–39.4)

**Table 4:** Number, percentage and different combinations of mixed seropositivity to vector-borne pathogens in the 300 dogs of the present study.

Pathogens	n (%; 95% CI)
***Ehrlichia* spp. + *Dirofilaria immitis***	12 (4; 2.1–6.9)
***Ehrlichia* spp. + *Anaplasma* spp. + *Dirofilaria immitis***	4 (1.3; 0.3–3.4)
***Anaplasma* spp. + *Dirofilaria immitis***	2 (0.7; −0.1–2.4)
***Ehrlichia* spp. + *Anaplasma* spp.**	1 (0.3; 0.01–1.8)
***Ehrlichia* spp. + *Anaplasma* spp. + *Dirofilaria immitis* + *Leishmania infantum***	1 (0.3; 0.01–1.8)
**Total**	20 (6.7; 4.1–10.1)

## Data Availability

All the data generated are described in the present article.
